# Supplementary material for: Mutation of GGMP Repeat Segments of Plasmodium falciparum Hsp70-1 Compromises Chaperone Function and Hop Co-Chaperone Binding
Source: Int J Mol Sci. 2021 Feb 23;22(4):2226. doi: 10.3390/ijms22042226 (PMC7926355; doi:10.3390/ijms22042226)
Supplement: Supplementary file 1 [file ijms-22-02226-s001.zip › IJMS Supplementary Data.docx]

**Supplementary Data**

**Table S1. Unique Hydrogen bonding in GGMP mutants**

| Contact residue 1 | Contact residue 2 | Length/Å | Region |
| --- | --- | --- | --- |
| **PfHsp70-1_G632_** | | | |
| Ala633(O) | Asn636(N) | 3.280 | C-terminal loop |
| Ala635(O) | Gly639(N) | 2.602 | C-terminal loop |
| Pro651(O) | Ser654(N) | 3.305 | C-terminal loop |
| Met627(O) | Gly630(N) | 3.128 | C-terminal loop |
| Ala616(N) | Gly626(O) | 2.561 | C-terminal loop |
| Leu623(O) | Gly626(N) | 3.426 | C-terminal loop |
| **PfHsp70-1_G648_** | | | |
| Pro647(O) | Asp651(N) | 2.790 | C-terminal loop |
| Ala648(O) | Asp651(N) | 3.151 | C-terminal loop |
| Gly645(O) | Ala649(N) | 3.319 | C-terminal loop |
| Met646(O) | Leu635(N) | 3.006 | C-terminal loop |
| Gly645(O) | Ala649(N) | 3.196 | C-terminal loop |
| Gly644(O) | Ala648(N) | 3.226 | C-terminal loop |
| Met646(O) | Met642(N) | 2.952 | C-terminal loop |
| Gly645(O) | Gly644(N) | 3.026 | C-terminal loop |
| **PfHsp70-1_G632-664_** | | | |
| Thr428 (O) | Leu468 (N) | 3.385 | L_3,4_ and L_5,6_ |
| Thr428 (N) | Ala430 (O) | 3.253 | L_1,2_ |
| Pro562 (O) | Glu566 (N) | 2.932 | Helix E |
| Asn561 (O) | Ile565 (N) | 2.617 | Helix E |
| Ile627 (O) | Ala650 (N) | 2.918 | C terminal loop |
| **PfHsp70-1_ΔG_** | | | |
| Ala404(O) | Val407(N) | 2.757 | L_1,2_ |
| Thr428(O) | Lys468(N) | 2.974 | L_3,4_ and L_5,6_ |
| **PfHsp70-1** | | | |
| Thr403(O) | Gly406(N) | 2.993 | L_1,2_ |
| Thr403(N) | Val407(O) | 3.038 | L_1,2_ |
| Val486(N) | Ile499(O) | 2.985 | β3 and β5 |
| Ala488(N) | Asn497(O) | 2.953 | β3 and β5 |
| Lys505(N) | Thr502(O) | 2.918 | Loop connecting SBDβ and SBDα |

*Contact atoms are shown in brackets

**Table S2: ATPase kinetics of PfHsp70-1, DnaK and their respective GGMP variants**

|  | *V_max_* (nmol/min/mg) [±SD] | *K_m_* (µM) [±SD] |
| --- | --- | --- |
| PfHsp70-1 | 29.93 [±1.6] | 364 [±3.5] |
| PfHsp70-1_G632_ | 35.72 [±2.9] | 1346 [±4.6] |
| PfHsp70-1_G648_ | 31.72 [±2.1] | 929 [±4.3] |
| PfHsp70-1_G632-664_ | 3.09 [±0.7] | 1085 [±7.5] |
| PfHsp70-1_∆G_ | 3.22 [±0,3] | 41.3 [±1.7] |
| DnaK | 8.39 [±1.2] | 1123.9[±5.9] |
| DnaK-G | 6.00 [±0.4] | 666 [±1.8] |

Table legends: **V_max_** – the maximum rate of the catalysis reaction; **K_m_** *-* is the substrate concentration at which the reaction rate is at half-maximum. At least three independent assays were carried out and results are shown as mean and standard deviations.

**Supplementary S3. Comparative peptide binding affinities of PfHsp70-1 and its GGMP variants**

| Protein | Nucleotide | K_D_ (M) [±S.D] |
| --- | --- | --- |
|  |  | **ANNNMYRR** |
| PfHsp70-1 | NN | 1.15 (±0.05) e^-8^ |
|  | ADP | 1.26 (±0.08) e^-8^ |
|  | ATP | 1.02 (±0.02) e^-7^ |
| PfHsp70-1_G632_ | NN | 1.03 (±0.3) e^-8^ |
|  | ADP | 2.09 (±0.09) e^-8^ |
|  | ATP | 1.64 (±0.04) e^-7^ |
| PfHsp70-1_G648_ | NN | 3.58 (±0.08) e^-9^ |
|  | ADP | 5.20 (±0.20) e^-9^ |
|  | ATP | 3.91 (±0.01) e^-8^ |
| PfHsp70-1_G632-664_ | NN | 1.50 (±0.50) e^-7^ |
|  | ADP | 1.88 (±0.08) e^-7^ |
|  | ATP | 2.18 (±0.08) e^-7^ |
| PfHsp70-1_∆G_ | NN | 1.17 (±0.07) e^-7^ |
|  | ADP | 1.78 (±0.08) e^-7^ |
|  | ATP | 6.92 (±0.02) e^-7^ |

**Table S4. Kinetics for PfHop interaction with PfHsp70-1 and its GGMP variants**

| Analyte | Ligand | Nucleotide | K_D_ (M) [±S.D] | *X*^2^ |
| --- | --- | --- | --- | --- |
| PfHsp70-1 | PfHop | NN | 9.11 (±0.1) e^-9^ | 0 |
|  |  | ADP | 1.58 (±0.0012) e^-8^ | 0.11 |
|  |  | ATP | 2.35 (±0.016) e^-7^ | 2.7 |
| PfHsp70-1_G632-664_ | PfHop | NN | 1.40 (±0.007) e^-6^ | 0.17 |
|  |  | ADP | 2.06 (±0.01) e^-6^ | 0.12 |
|  |  | ATP | 4.29 (±0.14) e^-6^ | 0.02 |
| PfHsp70-1_G648_ | PfHop | NN | 2.23 (±0.03) e^-8^ | 1.57 |
|  |  | ADP | 3.34 (±0.06) e^-8^ | 5.49 |
|  |  | ATP | 1.14 (±0.01) e^-7^ | 2.76 |
| PfHsp70-1_G632_ | PfHop | NN | 4.19 (±0.1) e^-8^ | 7.26 |
|  |  | ADP | 4.71 (±0.0011) e^-8^ | 1.84 |
|  |  | ATP | 2.37 (±0.8) e^-6^ | 2.04 |
| PfHsp70-1_∆G_ | PfHop | NN | 1.47 (±0.03) e^-6^ | 0.32 |
|  |  | ADP | 6.06 (±0.2) e^-7^ | 0.42 |
|  |  | ATP | 1.47 (±0.02) e^-6^ | 0.34 |

**Figure S1. Purification of PfHsp70-1 GGMP variants.** SDS-PAGE analysis for the expression of (A) PfHsp70-1_G632_; (B) PfHsp70-1_G648_; (C) PfHsp70-1_G632-664;_ (D) PfHsp70-1_ΔG_; (E) DnaK; and (F) DnaK-_G_. M represents the molecular weight marker (in kDa), P represents the pellet fraction, S: supernatant, FT: flow through, W: washes, E: elution fractions, C: control cells transformed with pQE30 vector, 0: preinduction and 6: 6 hrs post induction cell lysate.
